# Draft genome sequence data of *Oceanobacillus* sp. M60 isolated from soil in a conserved region of Sipadan Island, Sabah, Malaysia

**DOI:** 10.1128/mra.00420-26

**Published:** 2026-09-24

**Authors:** Ching Ching Wee, Mardani Abdul Halim, Hermin Pancasakti Kusumaningrum, Clemente Michael Wong Vui Ling, Zarina Amin

**Affiliations:** 1 Biotechnology Research Institute, Universiti Malaysia Sabah 60606, Kota Kinabalu, Sabah, Malaysia; 2 Biotechnology Study Program, Biology Department, Faculty of Sciences and Mathematics, Diponegoro University 95445, Semarang, Indonesia; California State University San Marcos, San Marcos, California, USA

**Keywords:** *Oceanobacillus*, soil microbiology, Sipadan Island, genomes

## Abstract

We present the draft genome sequence of *Oceanobacillus* sp. strain M60 isolated from soil collected in a conserved area of Sipadan Island, Sabah, Malaysia. It consists of 18 contigs totaling 4,590,102 bp, with a predicted total of 4,302 protein-coding genes, 6 rRNA genes, and 50 tRNA genes.

## ANNOUNCEMENT

*Oceanobacillus* species are rod-shaped, gram-positive bacteria belonging to the family Bacillaceae. The first reported species in the genus, *Oceanobacillus iheyensis*, is isolated from deep-sea sediment at a depth of 1,050 m on the Iheya Ridge and exhibits extreme halotolerance and alkaliphilic characteristics (1). As of 1 April 2026, a total of 36 *Oceanobacillus* species have been deposited into the National Center for Biotechnology Information (NCBI) database. In this study, *Oceanobacillus* sp. was isolated from the soil at Sipadan Island within the Sabah Parks Marine area situated in the Semporna District of Sabah, Malaysia, at latitude 4^o^7′0.33″ N and longitude 118^o^37′40.972″ E. Approximately 10 g of soil was collected from a sandy-calcareous substrate at a depth of ~15 cm below the ground surface. One gram of the soil sample was suspended in 9 mL of sterile phosphate-buffered saline (PBS) and serially diluted to 10^−5^. Subsequently, a 100 µL aliquot from the final dilution (10^−5^) was spread onto nutrient agar (NA) plates (HiMedia, India) and incubated under aerobic conditions at 25°C for 16 h. Pure cultures were obtained by streaking onto fresh NA plates, and then inoculated into nutrient broth, followed by incubation at 25°C for 24 h. Genomic DNA Buffer Set Kit (Qiagen, USA) was used to extract DNA from the bacteria following the manufacturer’s protocol. Whole-genome sequencing library of isolate M60 was prepared according to the manufacturer’s instructions using the Illumina DNA Prep Kit (Illumina, San Diego, USA) and sequenced on the Illumina NovaSeq 6000, 150 bp paired-end run, generating 13,368,098 reads. The raw sequencing reads were trimmed using Trimmomatic v0.40 (2) to obtain high-quality clean reads following slightly modified parameters (TruSeq3-PE-2.fa:2:30:10 LEADING:3 TRAILING:3 SLIDINGWINDOW:4:15 MINLEN:75). *De novo* genome assembly was carried out using Spades v4.2.0 (3), and assembly quality was assessed with QUAST v5.3.0 (4) using the default parameters. Genome completeness and contamination were estimated using CheckM v1.2.4 (5). Annotation of M60 was performed using the NCBI Prokaryotic Genome Annotation Pipeline (PGAP) v6.10 (6), with the “best-placed reference protein set” method in combination with GeneMarkS-2+ (7) (accessed 12 April 2025). In addition, genome annotation was performed using the Rapid Annotations using Subsystems Technology (RAST) Server (8) (https://rast.nmpdr.org/) with the RASTtk pipeline (9) on 25 May 2026 using default parameters.

**Fig 1:**
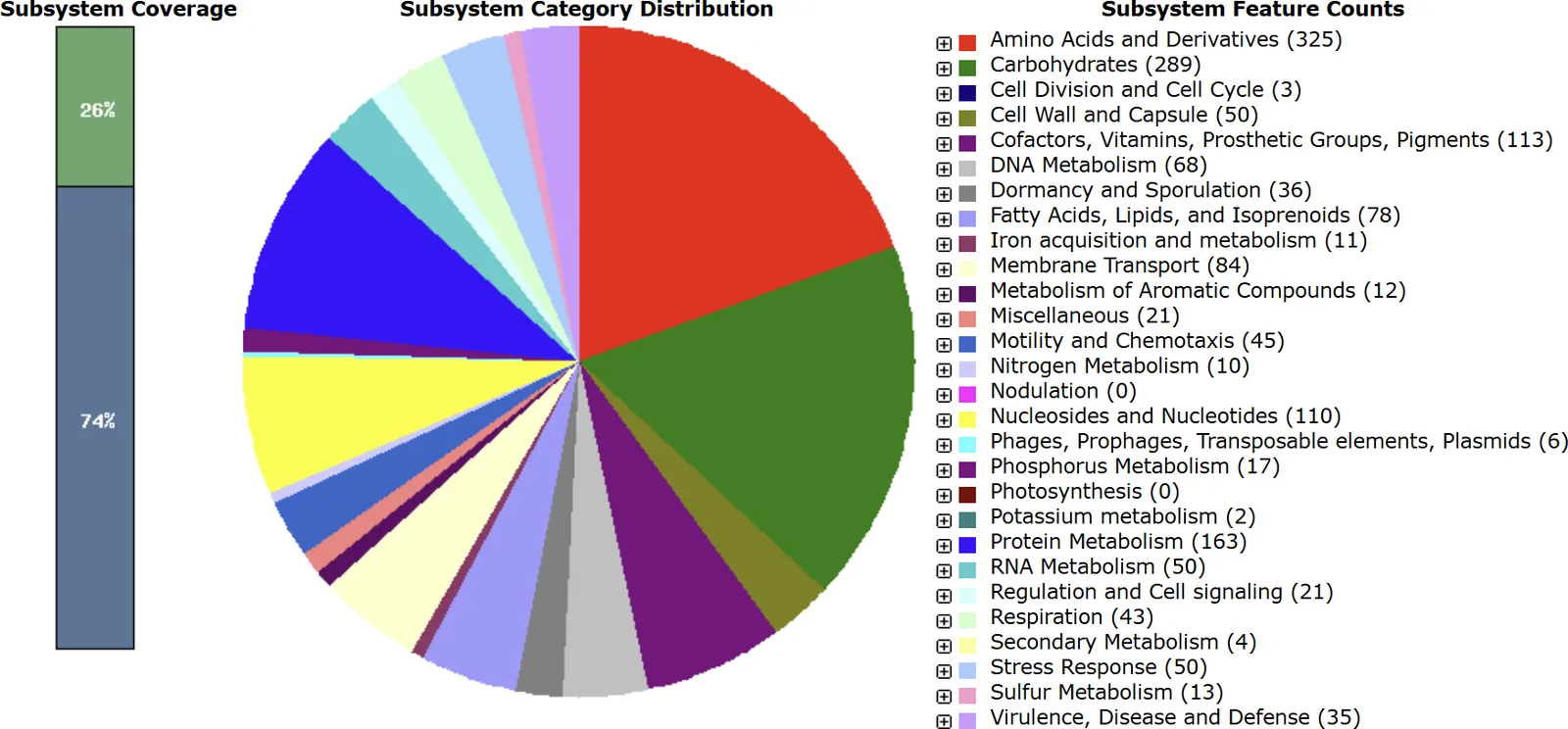
An overview of the subsystem statistics for *Oceanobacillus* sp. strain M60.

The assembly statistics are summarized in Table 1. CheckM analysis estimated genome completeness at 99.33% and contamination at 0.67%. Taxonomic classification of the assembled genome was performed using the GTDB-Tk Classify genomes tool (Galaxy Version 2.5.2+galaxy1) on the Galaxy Europe platform against the GTDB-Tk reference database (full release 220; 2024-10-28). The genome was placed within the genus *Oceanobacillus*, with *Oceanobacillus oncorhynchi* (GCF_000821065.1) identified as the closest genome based on average nucleotide identity (ANI) (99.03%). Functional annotation via the Rapid Annotations using Subsystems Technology (RAST) server revealed that amino acids and derivatives represented the most abundant subsystem category (Fig. 1), with branched-chain amino acids (79) and lysine, threonine, methionine, and cysteine (75) being the most represented subcategories.

**TABLE 1:** Genome features of the *de novo* assembled *Oceanobacillus* sp. strain M60 based on contig size ≥500 bp

Attribute	Value
Genome size (bp)	4,590,102
Number of contigs	18
Genome coverage	422×
GC content (%)	39.2
Largest contig (bp)	838,510
N50	539,981
L50	4
Total genes	4,414
Protein-coding genes	4,302
tRNAs	50
rRNA genes	6
5S rRNAs	3
16S rRNA	1
23S rRNAs	2

## Data Availability

This whole-genome shotgun project has been deposited in the National Center for Biotechnology Information (NCBI) under accession number JBSSNH000000000, BioProject ID accession number PRJNA990507, BioSample ID accession number SAMN36269618, and SRA accession number SRR25182454. The RAST annotation output is available on figshare at https://doi.org/10.6084/m9.figshare.32647011.
